# Over-the-Counter Bronchodilators Use Among Asthmatic Patients in Al-Medina Al-Monawwara

**DOI:** 10.7759/cureus.53026

**Published:** 2024-01-26

**Authors:** Anas Khalil, May M Almouteri, Samah F Alraddadi, Ebtesam A Abdullah, Rehab H Aljohani, Reem M Alhejaily

**Affiliations:** 1 Internal Medicine, Taibah University, Madinah, SAU; 2 Medicine, Taibah University, Madinah, SAU

**Keywords:** saba, inhaler, prescription, bronchodilator, over the counter

## Abstract

*Asthma* is a prevalent chronic disease that affects a significant number of individuals worldwide. Proper diagnosis and assessment of asthma patients are crucial before determining the appropriate inhaler for them. Many asthmatic patients self-medicate with over-the-counter (OTC) inhaled and orally taken bronchodilators, leading to overuse and serious adverse effects. This study aims to identify the extent of OTC bronchodilator usage in the Al-Medina Al-Monawwara region. and urge health authorities to address the issue and prevent potential side effects arising from the overuse of bronchodilators. This cross-sectional observational study was conducted among individuals with asthma residing in the Al-Medina Al-Monawwara region of Saudi Arabia. The study encompassed multiple hospitals. It was carried out between September 2021 and February 2023, utilizing a convenience sampling method. A total of 419 participants were included in the study; the majority of them, 362 (86.4%), reported being prescribed asthma inhalers at least once in their lives, while 57 (13.6%) always used asthma inhalers without a prescription. However, most of our participants reported having a prescription for their short-acting bronchodilators, with 46.3% always obtaining them with a prescription and the remaining obtaining them over the counter. The current study indicates that the use of OTC Bronchodilators increases the risk of significant ER visits; this observation is quite concerning as the increasing use of short-acting beta-agonists (SABA) might indicate less asthma control and more SABA overuse; further research is needed to address the issue of short-acting bronchodilators overuse.

## Introduction

Asthma is a prevalent chronic disease that affects over 300 million individuals globally, resulting in one out of every 250 deaths worldwide. [1] In Saudi Arabia, asthma is one of the most common chronic conditions, with a prevalence ranging from 15-25% across various regions [1-2]. Effective asthma management involves consistently using multiple inhaled medications, primarily inhaled corticosteroids (ICS) and bronchodilators [3].

Proper diagnosis and assessment of asthma patients are crucial before determining the appropriate inhaler for them. Inhaler medications should be prescribed by physicians rather than purchased over-the-counter (OTC) to avoid undertreatment and overtreatment of asthma. It is important to note that dispensing these medications without a prescription is illegal in some countries [3]. In Saudi Arabia, using short-acting bronchodilators with medical prescriptions is uncommon, possibly due to the availability of OTC inhalers that do not require a prescription. However, studies indicate that many asthmatic patients self-medicate with OTC inhaled and orally taken bronchodilators, leading to overuse and serious adverse effects such as poor asthma control, increased airway hyper-responsiveness, and even asthma-related mortality. Overuse of rescue inhalers is also linked to exacerbations, reduced productivity, poor quality of life, increased healthcare utilization, and under-treatment, ultimately increasing the disease burden on the healthcare system [3-5].

We observed inadequate data on using OTC bronchodilators among asthmatic patients in Saudi Arabia. This study aims to identify the extent of OTC bronchodilator usage among asthmatic patients in Al-Medina Al-Monawwara and identify the safety of using them as OTC.

## Materials and methods

This cross-sectional observational study was conducted among individuals with asthma residing in the Al-Medina Al-Monawwara region of Saudi Arabia. A total of 419 participants were included in the study; inclusion criteria were asthmatic Saudi and non-Saudi patients residing in the Al-Medina Al-Monawwara region.

The study encompassed multiple hospitals, including Ohud Hospital, King Fahad Hospital, Miqat Hospital, Yanbu General Hospital, and several primary healthcare centers. Participants less than 10 years old and non-asthmatic bronchodilator users were excluded. The study was carried out between September 2021 and February 2023, utilizing a convenience sampling method. We chose this sampling method because it is the most appropriate to reach the required sample, and that was through social media platforms; the sample size was calculated using Openepi software, and the minimum sample size required for this study was 384, assuming that the anticipated frequency is 50%, the margin of error is 5%, and the confidence interval is 95%.

The study's objectives were explained to the data collectors, who volunteered their efforts. Medical students and interns who volunteered to participate with us as data collectors interviewed participants and collected basic information such as age, gender, and educational level. The questionnaire was divided into three sections: socio-demographic data, health status-related factors, and bronchodilator use-related factors.

Participants were assured of their confidentiality, and their informed consent was obtained; for the children who were under 15 years old, we took consent from the guardian or his representative, while for those who were 15-18 years old, the consent from the patient and his guardian or his representative. Ethical approval was granted by the Directory of Health Affairs in Madinah (IRB142-2021).

The collected data were analyzed using IBM SPSS Statistics (Version 22). Using frequencies and numbers, participants were divided based on their socio-demographic factors, health status, and bronchodilator-related factors. Chi-square and one-way ANOVA analyses were employed to compare the distribution of bronchodilator category factors (namely, always prescribed, not always prescribed, the most time prescribed, and most time not prescribed) among participants based on their characteristics. To compare participants who reported prescribed bronchodilator use with those who reported no prescription of bronchodilator use, chi-square and t-tests were employed as appropriate. The level of statistical significance was defined as p≤0.05. 

## Results

Table 1 displays the socio-demographic characteristics of the study participants (n= 419). Most participants were aged less than 30 years (51.3%), while those aged ≥ 50 constituted 18.9% of the sample. Female participants accounted for 63.5% of the total, with male participants representing 36.5%. Approximately 37% of participants had received a university or higher education. The rest of the table displays the health status characteristics of the study participants (n= 419). The diagnosis of asthma was made before the age of 12 years for 46.3% of participants, and pulmonologists diagnosed asthma in 59.9% of participants. Approximately 53.2% of participants reported visiting a doctor for asthma-related issues. The most common symptom reported was breathing difficulty (7.2%), followed by dry cough (5%). Pulmonary function tests were conducted in 55.1% of participants. Among the participants, 19.6% perceived their condition as severe, and 7.2% as very severe. Approximately 72.3% of participants reported visiting the emergency department due to asthma, while 42.2% had been admitted to the hospital because of asthma.

**Table 1:** Demographic criteria and distribution of the studied participants by their health status-related factors (n=419) PFTs: Pulmonary Function tests

Socio-demographic	variable	n (%)
Age	10-29 years	215 (51.3)
	years 30-49	125 (29.8)
	≥ year 50	79 (18.9)
Gender	Male	153 (36.5)
	Female	266 (63.5)
Educational level	Less than secondary	130 (31.0)
	secondary	134 (32.0)
	University and higher	155 (37.0)
Age of Asthma diagnosis	≤ 12 years	194 (46.3)
	> 12 years	225 (53.7)
Diagnosis of asthma was done by:	Self-diagnosis	21 (5.0)
	Family physician	90 (21.5)
	Emergency department physician	57 (13.6)
	Pulmonologist	251 (59.9)
Regular doctor visits for follow-up regarding asthma symptoms.	Yes	223 (53.2)
	No	196 (46.8)
Pulmonary Function tests (PFTs) has been done at diagnosis.	Yes	231 (55.1)
	No	188 (44.9)
Symptoms experienced	Dry cough	21 (5.0)
	Wheezing	16 (4.0)
	Breathing difficulty	30 (7.2)
	Sleeping disorders	13 (3.1)
	Multiple symptoms	339 (80.9)
Participants perceive their condition as	Mild	149 (35.6)
	Moderate	158 (37.7)
	Severe	82 (19.6)
	Very severe	30 (7.2)
Emergency department visits	Yes	305 (72.3)
	No	116 (27.7)
Previous Hospital admissions related to asthma	Yes	177 (42.2)
	No	242 (57.8)

Table 2 illustrates the distribution of the participants in relation to bronchodilator use. The results indicate that a vast majority of the studied patients (86.4%) reported using asthma inhalers in the current and previous years. Moreover, 86.9% of the participants stated that they were prescribed asthma inhalers. Among those who reported using inhalers, 46.3% reported always buying them with a prescription, while 21% purchased inhalers without a prescription. Regarding specific bronchodilators, albuterol was the most commonly used inhaler (67.3%), whereas fluticasone/salmeterol was the least frequently reported (2.6%).

**Table 2:** Distribution of the studied participants based on their bronchodilator usage.

Factors	n (%)
Did you use an asthma inhaler during this year or last year?	
Yes	362 (86.4)
No	57 (13.6)
Have you ever received a prescription for an asthma inhaler?	
Yes	369 (86.9)
No	55 (13.1)
How do you obtain your asthma inhaler?	
Through a medical prescription	194 (46.3)
over-the-counter purchase	88 (21.0)
combination of both, prescription and over-the-counter methods	137 (32.7)
Which one of these bronchodilators you use	
albuterol only	282 (67.3)
budesonide/formoterol only	21 (5.0)
fluticasone/salmeterol only	11 (2.6)
Multiple inhalers	105 (25.1)

Distribution of the studied socio-demographic and health status-related factors by bronchodilator prescription (Table 3). Except for the age of asthma diagnosis, hospital admission because of asthma, and how patients see their condition, there have been statistically significant differences in socio-demographic and health-related factors between participants based on their prescription status. The most prevalent use of always-prescribed bronchodilators was among patients aged 10-29 years (49%), females (69.6%), patients diagnosed by Pulmonologist (70.6%), patients with multiple symptoms (86.1%), patients who reported visiting a doctor because of asthma symptoms (68.6%), and in those reported emergency department visits (76.3%). On the other hand, however, the most prevalent use of always without prescription bronchodilators was among male patients (53.4%), university graduate and highly educated patients ( 46.6%), patients who denied both doctor visits (73.9%) and hospital admissions 64.8%. 

**Table 3:** Distribution of socio-demographic and health status-related factors among the participants in the study, categorized by the method through which they acquire their bronchodilators.

Socio-demographic and health status-related factors	Bronchodilators use.				P value ≤ 0.05
						Always with prescription(n= 194)	Always over the counter (n= 88)	Usually with prescription(n= 61)	Usually over the counter(n= 76)
	Age years in categories										
	10-29	95 (49.0)	41 (46.6)	41 (67.2)		38 (50.0)	
30-49	55 (28.4)	34 (38.6)	15 (24.6)	21 (27.6)	
≥ 50	44 (22.7)	13 (14.8)	5 (8.2)	17 (22.4)	0.04*
Sex					
Male	59 (30.4)	47 (53.4)	19 (31.1)	28 (36.8)	
Female	135 (69.6)	41 (46.6)	42 (68.9)	48 (63.2)	0.002*
Educational level					
Less than secondary	70 (36.1)	15 (17.0)	25 (41.0)	20 (26.3)	
Secondary	62 (32.0)	32 (36.4)	23 (23.0)	26 (34.2)	
University and higher	62 (32.0)	41 (46.6)	22 (36.0)	30 (39.5)	0.02*
Age of Asthma diagnosis					
≤ 12 years	83 (42.8)	40 (45.5)	33 (54.1)	38 (50.0)	
> 12 years	111 (57.2)	48 (54.5)	28 (45.9)	38 (50.0)	0.41
Diagnosis of asthma was done by:					
Self-diagnosis	2 (1.0)	18 (20.5)	0 (0.0)	1 (1.3)	
Family physician	28 (14.4)	17 (19.3)	22 (36.1)	23 (30.3)	
Emergency physician	27 (13.9)	10 (11.4)	3 94.9)	17 (22.4)	
Pulmonologist	137 (70.6)	43 (48.9)	36 (59.0)	35 (46.1)	< .0001*
regular doctor visits for follow-up regarding asthma symptoms.					
Yes	133 (68.6)	23 (26.1)	41 (67.2)	26 (34.2)	
No	61 (31.4)	65 (73.9)	20 (32.8)	50 65.8)	< .0001*
Pulmonary Function tests done at diagnosis					
Yes	123 (63.6)	44 (50.0)	32 (52.5)	32 (42.1)	
No	71 (36.4)	44 (50.0)	29 (47.5)	44 (47.9)	0.01*
Participants perceive their condition as					
Mild	59 (30.4)	36 (40.9)	21 (34.4)	33 (43.4)	
Moderate	77 (39.7)	29 (33.0)	28 (45.9)	24 (31.6)	
Severe	40 (20.6)	18 (20.5)	9 (14.9)	15 (19.7)	
Very severe	18 (9.3)	5 (5.7)	3 (4.9)	4 (5.3)	0.42
Emergency department visits					
Yes	148 (76.3)	54 (61.4)	41 (67.2)	60 (78.9)	
No	46 (23.7)	34 (38.6)	20 (32.8)	16 (21.1)	0.03*
Previous Hospital admissions related to asthma.					
Yes	96 (49.5)	31 (35.2)	22 (36.1)	28 (36.8)	
No	98 (50.5)	57 (64.8)	39 (63.9)	48 (63.2)	0.06

**Figure 1:**
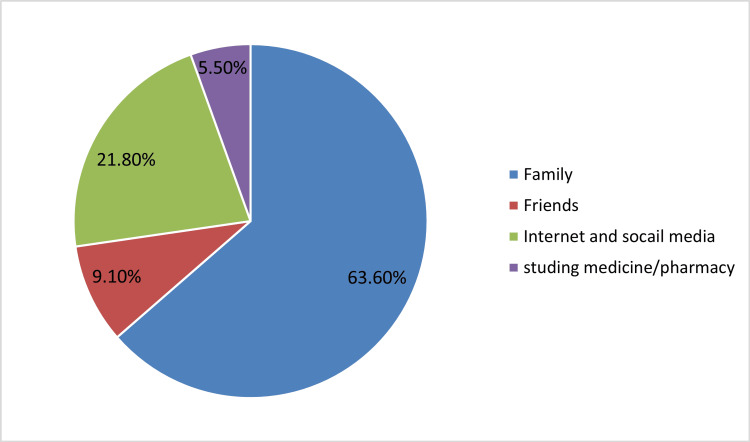
Source of knowledge among those who obtain their bronchodilators over the counter (n= 55)

Figure 1 displays the origin of information among the group of patients (n=55) who were not prescribed asthma inhalers. The majority of these patients reported that their family was the most common source of information (63.6%), followed by the internet and social media (21.8%) and friends (9.1%). The least common source of knowledge was self-education by patients themselves(5.5%).

Table 4 displays the distribution of factors related to bronchodilator usage based on the method through which bronchodilators are acquired. A highly significant statistical difference was observed between prescription status and the mean number of weekly and daily usage of bronchodilators, where patients who always reported over-the-counter bronchodilator use had a higher mean value (14.6 ± 13.4 and 3.2 ± 3.5 for weekly and daily usage, respectively). Furthermore, the percentage of patients who always reported over-the-counter bronchodilator use was higher among those who obtained bronchodilators from a private pharmacy (77.3%), those who reported that the pharmacist did not inquire about the cause of use (72.7%), did not describe the correct usage for them (79.4%) and did not ask them about the use of other medications (83%), "Among patients who never needed systemic corticosteroids..." (72.7%).

**Table 4:** Distribution of factors related to bronchodilator usage based on the method through which bronchodilators are acquired.

Bronchodilators use related factors	Bronchodilators prescription				P value≤ 0.05
	Always with prescription (n= 194)	Always over the counter (n= 88)	Usually with prescription (n= 61)	Usually over the counter (n= 76)	
						mean frequency of albuterol usage					
Per week	7.1 ± 7.8	14.6 ± 13.4	5.1 ± 6.3	8.3 ± 8.9	< .0001*
Per day	1.5 ± 1.3	3.2 ± 3.5	2.0 ± 1.5	1.9 ± 1.7	< .0001*
Side effects were observed or reported following usage.					
Yes	95 (49.0)	47 (53.4)	26 (42.6)	34 (48.2)	
No	99 (51.0)	41 (46.6)	35 (57.4)	42 (51.8)	0.54
Locations where bronchodilators are obtained					
Private pharmacy	25 (12.9)	68 (77.3)	23 (37.7)	59 (77.6)	
Hospital pharmacy	169 (87.1)	20 (22.7)	38 (62.3)	17 (22.4)	< .0001*
During the acquisition of albuterol, did the pharmacists inquire about the reason for using this medication?					
Yes	70 (36.1)	24 (27.3)	24 (39.3)	26 (34.2)	
No	124 (63.9)	64 (72.7)	37 (60.7)	50 (65.8)	0.4
During the process of obtaining albuterol, did the pharmacist provided instructions on the proper technique and ensured that the patient was knowledgeable about how to use their bronchodilator?					
Yes	93 (47.9)	19 (21.6)	21 (34.4)	31 (40.8)	
No	101 (52.1)	69 (79.4)	40 (65.6)	45 (59.2)	< .0001*
During the acquisition of albuterol, did the pharmacist inquire about the concurrent use of other medications					
Yes	67 (34.5)	15 (17.0)	12 (19.7)	18 (23.7)	
No	127 (65.5)	73 (83.0)	49 (80.3)	58 (76.3)	0.01*
concurrent use of other medications alongside albuterol					
Yes	57 (29.4)	20 (22.7)	15 (24.6)	24 (31.6)	
No	137 (70.6)	68 (77.3)	46 (75.3)	52 (68.4)	0.53
Ever needing systemic corticosteroids for asthma control					
Yes	78 (40.2)	24 (27.3)	14 (23.0)	23 (30.3)	
No	116 (59.8)	64 (72.7)	47 (77.0)	53 (69.7)	0.03*

## Discussion

Most of our participants reported having a prescription for their short-acting bronchodilators, with 46.3% always obtaining them with a prescription. While 32.7% report that they sometimes use their short-acting bronchodilators with a prescription and sometimes purchase OTC, the remaining 21% report that they always purchase OTC. Our results align with a cross-sectional study done in December 2022, in which 502 patients from seven sites across Saudi Arabia showed SABA overprescription was widespread, with nearly a quarter of patients purchasing SABA OTC [6].

Most OTC bronchodilator users report fewer hospital admissions and doctor visits, but they have significant ER visits, which may indicate a correlation between using OTC bronchodilators and worse asthma outcomes. This finding contrasts with a study in Australia in 2011, which showed no association between OTC SABA medication use and worse asthma outcomes [7]. However, new studies do not recommend the use of SABA alone; using SABA alone was found to increase the risk of asthma-related death and asthma attacks, so ICS is used whenever SABA is needed [1]. In addition, a previous study showed that urgent doctor visits, ER visits, and hospitalization were seen among reliever-only users [8].

Also, another finding of the current study showed that the number of patients who consistently reported OTC bronchodilator use was higher among those who never used systemic corticosteroids. However, a previous study showed that SABA overuses are more likely to need a course of systemic corticosteroids [5]. This discrepancy may be because the patients who purchased OTC SABA tend to have milder asthma symptoms.

Moreover, our study showed that the overuse of bronchodilators was significantly higher among those who report OTC use; this observation is quite concerning as the increasing use might indicate less asthma control. A study done in Sweden in April 2020 showed that SABA overuse was associated with increased risks of exacerbation and mortality [9]. Our findings might also indicate poor asthma control among participants, as stated by another study done in November 2020, in which the authors concluded that the mean annual short-acting bronchodilator purchases were significantly higher in the partially controlled and uncontrolled Asthma patients [10].

Interestingly, our study showed no significant difference in the side effects experienced among the OTC group and the prescription one, even though overuse of bronchodilators was significantly higher among OTC users. Those findings contradict the conclusions of a systematic review conducted in November 2018, which concluded a slight increase in some adverse events for participants using higher doses of β2-agonists [11].

We also found an association between the use of prescribed SABA and the pharmacist's explanation of the correct use of the inhaler. This finding needs to be addressed and utilized to improve the outcomes of asthma treatment, and the explanation of the correct inhaler technique needs to be encouraged even if they were purchased over the counter, according to a recent systemic review done in September 2022, a better inhalation technique was associated with better asthma outcomes [12].

For those who obtained their short-acting asthma inhalers OTC, the most frequent source of knowledge found in our study was family members; they recommended using SABA. This finding suggests a significant gap in knowledge among our study participants regarding the latest asthma management updates; short-acting bronchodilator monotherapy is no longer recommended [13]. An educational program targeting the general population should be implemented, addressing the latest asthma management updates and bronchodilator overuse.

It is important to acknowledge certain limitations in our research, including convenience sampling, self-reported data, which may lead to self-reporting bias, a non-validated questionnaire, and the study design as a cross-sectional study. Even though this research has global implications, our study has critical educational messages for doctors who prescribe bronchodilators and pharmacists who dispense them. Moreover, prescribed bronchodilators by doctors would result in an improvement in the quality of care.

Asthma control worldwide is suboptimal; we need to take a multidisciplinary approach and engage physicians and pharmacists so that the issues we find are identified at all healthcare levels and at any point that patients seek treatment. If these bronchodilators continue to be OTC, further information should be obtained to determine whether OTC availability is a risk factor for asthma morbidity and mortality.

## Conclusions

In summary, 46.3% of the participants always obtained their asthma inhaler with a prescription. The current study indicates that using OTC bronchodilators increases the risk of significant ER visits, which may indicate a correlation between the use of OTC bronchodilators and worse asthma outcomes. In addition, the overuse of bronchodilators was significantly higher among those who reported OTC use. This finding might indicate less asthma control. Further study is needed to confirm these findings. Inhaler medications should be prescribed by physicians rather than purchased OTC to avoid uncontrolled asthma and worse asthma outcomes, so further studies are required to approve this finding. It is important to note that the questionnaire of the current study is non-valid; further studies are recommended using a valid and reliable one.
